# decompTumor2Sig: identification of mutational signatures active in individual tumors

**DOI:** 10.1186/s12859-019-2688-6

**Published:** 2019-04-18

**Authors:** Sandra Krüger, Rosario M. Piro

**Affiliations:** 1 Institute of Computer Science and Institute of Bioinformatics, Freie Universität Berlin, Berlin, Germany; 2 Institute of Medical Genetics and Human Genetics, Charité – Universitätsmedizin Berlin, Berlin, Germany; 3 German Cancer Consortium (DKTK) partner site Berlin, and German Cancer Research Center (DKFZ), Heidelberg, Germany

**Keywords:** Somatic mutations, Mutational processes, Mutational signatures, Signature refitting, Decomposition of tumor genomes

## Abstract

**Background:**

The somatic mutations found in a tumor have in most cases been caused by multiple mutational processes such as those related to extrinsic carcinogens like cigarette smoke, and those related to intrinsic processes like age-related spontaneous deamination of 5-methylcytosine. The effect of such mutational processes can be modeled by mutational signatures, of which two different conceptualizations exist: the model introduced by Alexandrov et al., *Nature* 500:415–421, 2013, and the model introduced by Shiraishi et al., *PLoS Genetics* 11(12):e1005657, 2015. The initial identification and definition of mutational signatures requires large sets of tumor samples.

**Results:**

Here, we present decompTumor2Sig, an easy to use R package that can decompose an individual tumor genome into a given set of Alexandrov-type or Shiraishi-type signatures, thus quantifying the contribution of the corresponding mutational processes to the somatic mutations identified in the tumor. Until now, such tools were available only for Alexandrov signatures. We demonstrate the correctness and usefulness of our approach with three test cases, using somatic mutations from 21 breast cancer genomes, from 435 tumor genomes of ten different tumor entities, and from simulated tumor genomes, respectively.

**Conclusions:**

The decompTumor2Sig package is freely available and has been accepted for inclusion in Bioconductor.

**Electronic supplementary material:**

The online version of this article (10.1186/s12859-019-2688-6) contains supplementary material, which is available to authorized users.

## Background

**Fig. 1:**
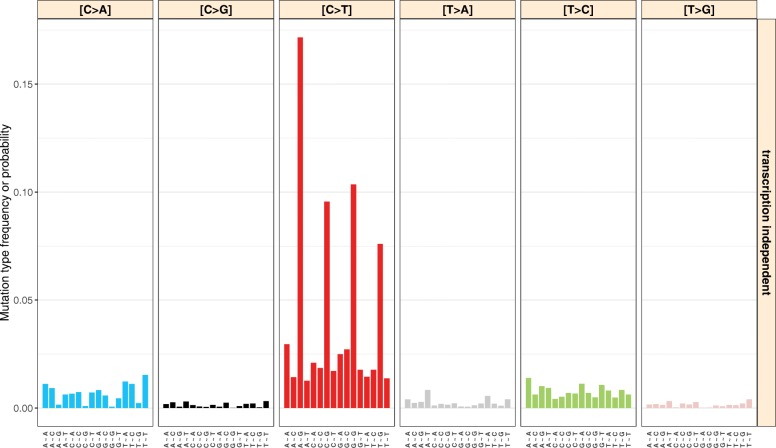
The Alexandrov model of mutational signatures. The example signature shown here represents mutations caused by spontaneous deamination of 5-methylcytosine (COSMIC signature 1) and uses one flanking base in each direction of the mutated base. The location of mutations with respect to the transcription strand is not taken into consideration. The data were obtained from COSMIC at http://cancer.sanger.ac.uk/cosmic/signatures

**Fig. 2:**
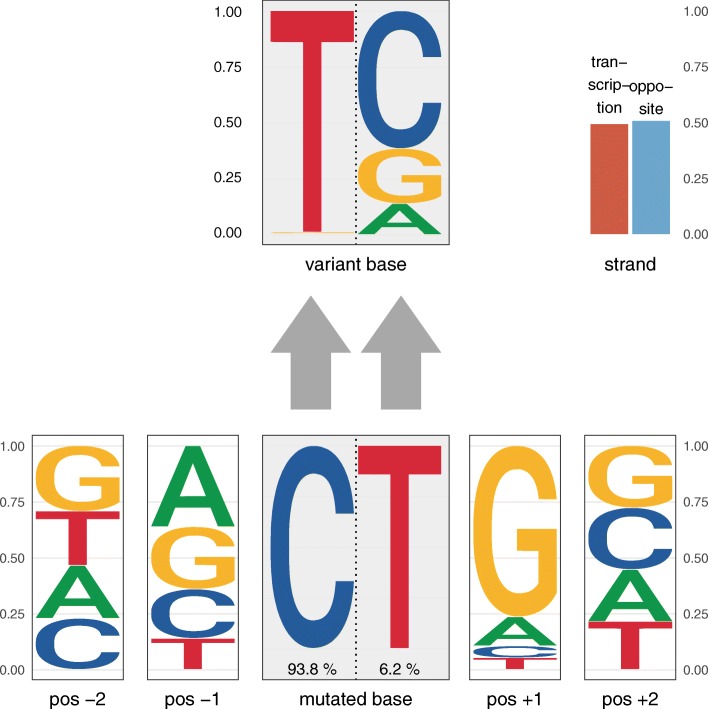
The probabilistic Shiraishi model of mutational signatures. The example signature shown here uses two flanking bases in each direction and was obtained from 435 tumor genomes. It likely represents mutations caused by spontaneous deamination of 5-methylcytosine. The corresponding mutation frequencies are shown in Table 1. In contrast to pmsignature’s graphical representation of Shiraishi-type signatures (see Figure S6 in Additional file 1), decompTumor2Sig’s representation of the mutation frequency data uses sequence logos for the flanking bases and the variant bases (with the heights of the bases being proportional to their probability/frequency). The mutated bases (C or T) are represented next to each other and their respective frequency is indicated below. This side-by-side representation of the mutated bases allows to distinguish the probabilities of variant bases (on top) according to the mutated base. Transcription strand bias (if information on transcription direction is used) is shown in the upper right corner

The mutational processes responsible for the somatic mutations observed in tumor samples can significantly vary not only between tumor types but also among the individual cancers within a tumor class. A mutational process can be represented by a so called “mutational signature” [1–3] which reflects the occurrences of base changes within their sequence contexts (i.e., in dependence on their flanking bases). The age-related mutations initiated by spontaneous deamination of 5-methylcytosine, for example, regard cytosine-to-thymine (C >T) transitions in the context of CpGs (because those are methylated; see Figs. 1 and 2, and Table 1). Other characteristic mutation patterns are known for exogenous mutagenic factors such as UV light and cigarette smoke (see [4] for a review). (Here and in the entire manuscript, we will consider only single nucleotide variants although we generically speak of mutations).

**Table 1:** Mutation frequencies according to the probabilistic Shiraishi model of mutational signatures, exemplified by the 6+4∗4+2=24 parameters of the signature depicted in Fig. 2

Nucleotide change (central base)						Flanking bases					Transcription strand	
C >A	C >G	C >T	T >A	T >C	T >G	Position	A	C	G	T	plus strand	minus strand
0.004	0.006	0.928	0.009	0.038	0.015	-2	0.237	0.228	0.293	0.242	0.493	0.507
						-1	0.362	0.220	0.279	0.139		
						+1	0.131	0.053	0.764	0.052		
						+2	0.232	0.277	0.277	0.214		

Each parameter is the fraction of mutations caused by the mutational process which exhibit the respective characteristic. For instance, 92.8% of the changes are C >T and there is next to no transcriptional bias (compare to Fig. 2)

### Alexandrov signatures

There are two conceptualizations of mutational signatures. The model first described by Alexandrov et al. [1, 2] separately counts all possible nucleotide triplets whose central base is mutated, describing, for example, the most frequent mutations caused by spontaneous deamination of 5-methylcytosine as A[C >T]G, C[C >T]G, G[C >T]G, and T[C >T]G. Given that there are a total of 6×4×4=96 possible triplet mutations (when removing the redundancy due to the reverse complement strand and considering only single nucleotide variants), each “Alexandrov signature”, as we call it, consists of 96 mutation probabilities that indicate which of the changes are occurring most frequently due to the mutational process it describes. As an example, see Fig. 1 which represents the spontaneous deamination of 5-methylcytosine.

### Shiraishi signatures

The second model [3] describes probabilistic mutational signatures in analogy to the way we model transcription factor binding motifs, i.e., considering the single bases of the motif as independent. Using this approach, the most frequent changes due to spontaneous deamination of 5-methylcytosine can be described as C >T transitions followed by a base that with a very high probability is a guanine (G), and preceded by any of the four bases with approximately equal probability (see Fig. 2 and Table 1). Instead of 96 parameters, a “Shiraishi signature”, as we call it, requires only 6+4+4=14 parameters when considering nucleotide triplets [3]: the frequencies of the six possible base changes at the center and the frequencies of the four possible bases of each of the flanking nucleotides. The major advantage of this significant reduction of parameters is that the signatures can be more easily extended to more flanking bases (e.g., two flanking bases in each direction as shown in Fig. 2), and the incorporation of the transcription-strand direction requires only two additional parameters instead of doubling the number of parameters.

As an example, Table 1 shows the parameters (equivalent to probabilities) of the Shiraishi signature for spontaneous deamination of 5-methylcytosine using a sequence context of five bases (with the altered base in the center) and taking transcription strand into consideration. Since the possible base changes, the individual flanking bases, and the transcription strand are treated as independent, the probabilities for each of them sums to 1. For more details, we refer to the original paper [3].

To summarize, while the Alexandrov model takes the full dependency between mutated nucleotides and their directly neighboring flanking bases into account, the simplified Shiraishi model treats mutated nucleotides and flanking bases as independent features of the signature. Due to this relaxed parameterization, however, the Shiraishi model is by definition unable to fully capture the existing relationships between the different features of a signature, such as the relationship between strand bias and sequence context, or between a specific nucleotide change and the following base [5]. Specific recurrent somatic mutations of polymerase Pol *ε*, for example, have been associated with a high frequency of T[C >A]T mutations, a high frequency of T[C >T]G mutations and some additional T[T >G]T mutations (Alexandrov signature no. 10 [2]). The corresponding Shiraishi signature would merely report high frequencies of guanines and thymines at the third base of the triplet, but not that guanines are associated with a cytosine-to-thymine (C >T) change and thymines with cytosine-to-adenine (C >A) and thymine-to-guanine (T >G) changes.

### Mutational signatures in individual tumor samples

The initial discovery and construction of mutational signatures (de novo signature inference) requires a large amount of tumor samples, such that regular patterns can be identified [1–3] using techniques such as non-negative matrix factorization, expectation–maximization, or probabilistic methods (see Baez-Ortega and Gori for a review [5]). This is impractical in a clinical setting, where each cancer patient is diagnosed individually. However, once accurate signatures have been defined, they can be used to evaluate their contribution to the mutational load in individual tumor samples (signature refitting). This helps to assess which mutational processes were likely involved in the development of the tumor—as has been demonstrated for Alexandrov signatures [6, 7]—and may be of clinical relevance, for instance, when they hint at a DNA repair deficiency, because DNA repair mechanisms significantly affect the response to cytotoxic treatments [8].

Here, we present a user-friendly R package, called decompTumor2Sig, that can be used to evaluate the contribution of Shiraishi signatures to the somatic mutations found in an individual tumor, allowing larger sequence contexts to be taken into consideration than with Alexandrov signatures. (In addition, the package can just as well be used for Alexandrov signatures, but we will discuss mostly Shiraishi signatures here).

## Methods

### Contribution of signatures to individual tumor samples

To derive the influence of a given set of Shiraishi signatures on the generation of the mutational catalog (i.e., the set of somatic mutations) of an individual tumor sample, the decompTumor2Sig package takes the same quadratic programming approach used by Lynch for Alexandrov signatures [7].

Let *g* be the tumor genome, described in terms of fractions of somatic mutations that have specific nucleotide changes, flanking bases and transcription strands. The representation of the tumor genome is thus identical to the representation of the single signatures as exemplified in Table 1. Let further **S** be a *P*×*K* matrix, with each column being one of *K* signatures composed of *P* parameters (here: *P*=6+4×4+2=24 for a total of four flanking bases, two in each direction, and two additional parameters for the transcription strand). The goal is to determine a vector *w* of weights *w*_*s*_ (Alexandrov et al. [1, 2] called them “exposures”) which indicates how strongly each signature *s*∈(1,*K*) contributed to the mutation load of the tumor, i.e., what fraction of the somatic mutations in *g* were caused by *s*. Of course, we would like to have **S***w*≈*g* with as little error as possible. We therefore can solve the following problem:

$$\begin{array}{@{}rcl@{}} {\begin{aligned} \text{minimize} & (g-\mathbf{S}w)^{T}(g-\mathbf{S}w) = g^{T}g - g^{T}\mathbf{S}w - (\mathbf{S}w)^{T}g + (\mathbf{S}w)^{T}\mathbf{S}w \\ & \mathrm{subject~to} \sum^{K}_{s=1} w_{s} = 1, w_{s} \ge 0 \\  \end{aligned}} \end{array} $$

This is equivalent to minimizing the squared error *ε*^*T*^*ε* because *g*=**S***w*+*ε*. Since *g*^*T*^*g* is constant and (**S***w*)^*T*^*g*=*g*^*T*^**S***w*, we can simplify the problem as:

$$\begin{array}{@{}rcl@{}} \text{minimize} & \,-\, g^{T}\mathbf{S}w \,+\, \frac{1}{2}w^{T}\mathbf{S}^{T}\mathbf{S}w\quad \!\!\! \mathrm{subject~to} &\! \sum^{K}_{s=1} w_{s} \,=\, 1, w_{s} \!\ge\! 0 \\  \end{array} $$

We solve this classical quadratic programming problem using the R package quadprog [9] and thus compute the contributions of the given Shiraishi signatures to the overall mutation load of the tumor.

### Positive definiteness of **S**^*T*^**S**

The R package quadprog implements the method of Goldfarb and Idnani [10, 11] for solving quadratic programming problems of the form $\text {min}\left (-d^{T}b + \frac {1}{2}b^{T}\mathbf {D}b\right)$ with a set of constraints described by *A*^*T*^*b*≥*b*_0_. For this method, the matrix **D**, which in our case corresponds to **S**^*T*^**S** (see above), needs to be positive definite, i.e., the scalar *b*^*T*^**D***b* (here: *w*^*T*^**S**^*T*^**S***w*) needs to be positive for every non-zero column vector *b*.

**Fig. 3:**
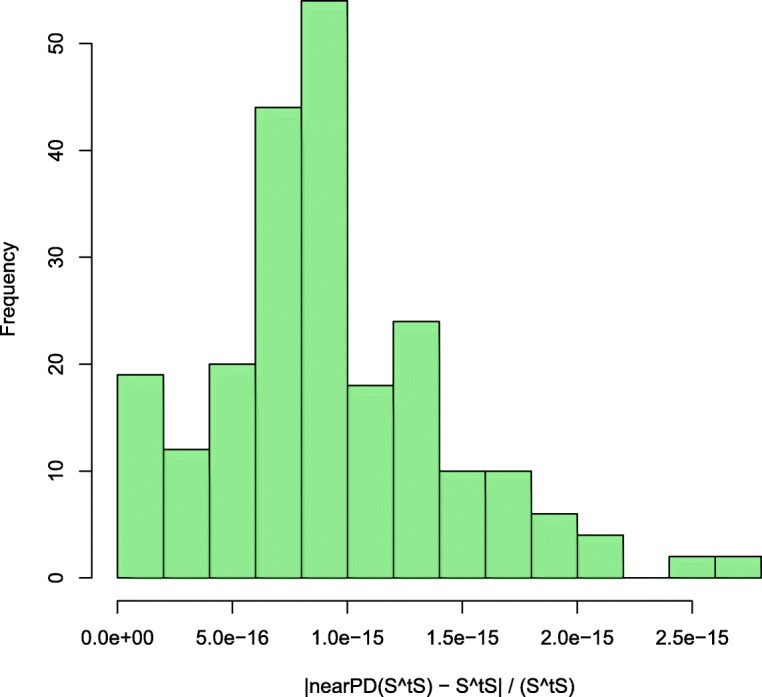
Difference between approximated and original **S**^*T*^**S**. The histogram shows that the absolute difference between the matrix elements of the approximated matrix and the original matrix (i.e., |nearPD(**S**^*T*^**S**)_*ij*_−(**S**^*T*^**S**)_*ij*_|) is only a negligible fraction of the corresponding element of the original matrix (**S**^*T*^**S**)_*ij*_. Data collected from the decomposition of 44 tumors with 15 signatures

As this is not the case for all signature matrices **S**, we convert **S**^*T*^**S** to its nearest positive definite matrix using the nearPD function provided by the R package Matrix. As illustrated by Fig. 3, the difference between the approximated, positive definite matrix and the original **S**^*T*^**S** is negligible. For 44 tumor decompositions with 15 signatures, the individual matrix elements of the approximated matrix diverged by a fraction less than 3*e*^−15^ of the original value, i.e., |nearPD(**S**^*T*^**S**)_*ij*_−(**S**^*T*^**S**)_*ij*_|<3*e*^−15^×(**S**^*T*^**S**)_*ij*_ for all matrix elements *ij*.

### Variance explained by subsets of signatures

In order to calculate the variance of the somatic mutation patterns of a tumor which can be explained by a given subset of signatures (or the complete set of signatures) after decomposition of the tumor genome, for the Alexandrov model decompTumor2Sig determines the coefficient of determination *R*^2^, i.e., the explained variance, as follows

$$\begin{array}{@{}rcl@{}} R^{2} = 1 - \frac{\text{Var}(g-\hat{g})}{\text{Var}(g)}= 1 - \frac{\sum^{P}_{i=1}{(g_{i} - \hat{g}_{i})^{2}}}{\sum^{P}_{i=1}{(g_{i} - \bar{g})^{2}}} \end{array} $$

where *g* is the observed mutational load and $\hat {g} = \mathbf {S}w$ is the mutational load predicted by the decomposition model, both described in terms of the 96 parameters which quantify the mutation frequencies of the triplet-based mutation categories.

Here, the numerator $\sum ^{P}_{i=1}{\left (g_{i} - \hat {g}_{i}\right)^{2}}$ is the residual sum of squares (RSS), or squared error, and the denominator $\sum ^{P}_{i=1}{\left (g_{i} - \bar {g}\right)^{2}}$ can be interpreted as the deviation of the observed parameters from a “flat” tumor genome with uniform mutation frequencies. Indeed, for the Alexandrov model, with its 96 mutation frequencies whose sum is 1, a flat tumor genome would have a uniform mutation frequency of 1/96 for each mutation category, which is precisely the mean mutation frequency $\bar {g}$. This is equivalent to having one model feature with 96 possible states.

For the Shiraishi model, however, a flat tumor genome of uniform mutation frequencies cannot be described by $\bar {g}$ because the features that compose the model are independent from each other and have different numbers of possible states—six for the base change, four for flanking bases, and two for the transcription-strand direction. Therefore, a flat tumor genome should have a mutation frequency of 1/6 for each possible base change, 1/4 for each possible flanking base, and 1/2 for each possible transcription-strand direction. We therefore define the explained variance for the Shiraishi model as

$$\begin{array}{@{}rcl@{}} R^{2} = 1 - \frac{\text{Var}\left(g-\hat{g}\right)}{\text{Var}(g)} = 1 - \frac{\sum^{P}_{i=1}{\left(g_{i} - \hat{g}_{i}\right)^{2}}}{\sum^{P}_{i=1}{\left(g_{i} - {g}^{*}_{i}\right)^{2}}} \end{array} $$

where the uniform, flat tumor genome *g*^∗^ is defined as exemplified in Table 2.

**Table 2:** Uniform mutation frequencies according to the probabilistic Shiraishi model of mutational signatures, exemplified by the same 6+4∗4+2=24 parameters as in Table 1

Nucleotide change (central base)						Flanking bases					Transcription strand	
C >A	C >G	C >T	T >A	T >C	T >G	Position	A	C	G	T	Plus strand	Minus strand
1/6	1/6	1/6	1/6	1/6	1/6	-2	1/4	1/4	1/4	1/4	1/2	1/2
						-1	1/4	1/4	1/4	1/4		
						+1	1/4	1/4	1/4	1/4		
						+2	1/4	1/4	1/4	1/4		

### Implementation and usage of decompTumor2Sig

The decompTumor2Sig package provides several functions to load tumor genome data from standard Variant Call Format (VCF) and Mutation Position Format (MPF) files, or to convert it from a VRanges object (Bioconductor package VariantAnnotation [12]) or from preprocessed data (“mutation feature data”) that has been loaded using Shiraishi et al.’s pmsignature package [3].

Likewise, Shiraishi signatures can be loaded either from flat files, or extracted from the preprocessed “estimated parameters data” obtained from pmsignature. Additionally, Alexandrov signatures can be downloaded directly from the COSMIC [13] website at http://cancer.sanger.ac.uk/cosmic/signatures, or loaded from a flat file that follows the same format. Alexandrov-type signatures can also be converted to Shiraishi-type signatures:

signatures_s <- convertAlexandrov2 Shiraishi(signatures_a)

Once the signatures and one or more tumor genomes have been loaded, decomposing each individual tumor according to the given signatures is straight forward and requires a single command:


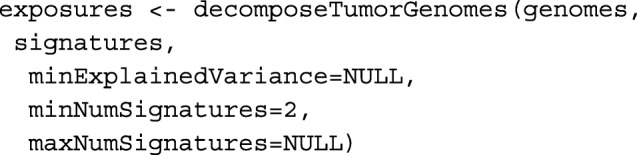


The function call returns the contributions, or exposures, of the single signatures to the overall mutation load of the individual tumors.

By default, all signatures are used for the decomposition, but if desired, only subsets of the given signatures can be considered, for example to explain a minimum fraction of the variance of the mutational patterns of a tumor. In this case, the function decomposeTumorGenomes will test all combinations of minNumSignatures to maxNumSignatures signatures (default: from 2 to the complete set), until a subset of signatures explains at least minExplainedVariance of the variance. This smallest subset of signatures is determined individually for each tumor genome, and the corresponding exposures are returned. Alternatively, users may use the parameter maxNumSignatures to explicitly specify the size of the subset of signatures to be used without requiring a minimum explained variance (minExplainedVariance=NULL).

To better evaluate how many signatures will be necessary to explain a certain variance, the function plotExplainedVariance can be used to plot the explained variance as a function of the number of used signatures. Further functions allow to plot the signatures (as the examples in Figs. 1 and 2) and the decomposed contributions, i.e., the computed exposures.

### Mapping between different sets of signatures

In some cases it might be desirable to map one set of signatures to another set of a different origin. A user might, for instance, have a set of Shiraishi signatures and want to know to which Alexandrov signatures from COSMIC they are likely related. To make different types of signatures comparable, we have—apart from the possibility to convert Alexandrov-type signatures to the Shiraishi model (see above)—implemented a set of functions which allow to

- “downgrade” Shiraishi-type signatures by reducing the number of flanking bases or discarding information on the transcription-strand direction (function downgradeShiraishiSignatures),
- measure the distances between a given signature (of Shiraishi or Alexandrov type) and each of a set of signatures of the same format (function determineSignatureDistances), where various distance metrics can be used, and
- determine a mapping from one set of signatures (of Shiraishi or Alexandrov type) to another set of signatures of the same format (function mapSignatureSets).

## Results and discussion

### Estimation of accuracy for actual tumor data

To estimate the accuracy with which we can determine the contribution of Shiraishi-type mutational signatures to the mutation load of an individual tumor, we proceed as follows:

1. For a given set of *T* tumors, using the R package pmsignature [3] we collectively derive a set of Shiraishi signatures **S** and their corresponding contributions/weights *w*^(*t*)^, *t*∈(1,*T*) to the tumor genomes. We take these computed weight vectors as “truth” set.
2. We take either each single tumor out of the set (leave-one-out test) or, for larger sets, a randomly chosen subset (test set). The remaining tumors are used to collectively recompute signatures *S*^′^ as in 1.
3. For each individual tumor *t*^∗^ to be tested, we estimate the contributions/weights $w'^{(t^{*})}$ using our tool (decompTumor2Sig) on the test signatures *S*^′^, i.e., on the signatures that were derived without mutation data from *t*^∗^. Note, this constitutes a realistic application where one has a given set of signatures and wants to apply them to a novel tumor sample.
4. We determine a 1:1 mapping between the test signatures *S*^′^ and the original signatures **S** (minimum Frobenius distance between the signature matrices), so that we can compare the contributions $w'^{(t^{*})}$, estimated by decompTumor2Sig, to the “true” contributions originally computed using pmsignature in step 1.

Please note that with this procedure we actually underestimate the accuracy of decompTumor2Sig because during a de novo signature inference, as is done by pmsignature, exposures are determined along with the the signatures themselves, leading to a much higher number of parameters to be estimated. Signature refitting, as we do here, is a comparably limited problem and gives more accurate exposures, as we demonstrate below. Nonetheless, we will use the evaluation procedure outlined above because it best reflects the general use case where one has a novel tumor sample that has not been included in the de novo signature inference.

### Evaluation for actual tumor data

We performed two tests to show that our tool can effectively decompose the mutation catalog of an individual tumor and determine the relative contributions of a given set of Shiraishi signatures:

1. We performed a leave-one-out cross validation using somatic mutations from a set of 21 breast cancer genomes [14]. Given the limited size of the dataset, we decomposed the tumors into four Shiraishi signatures, as was done for breast cancer by Shiraishi et al. [3].
2. We performed a second test using 435 tumor genomes with at least 100 somatic mutations from ten different tumor entities [2] (acute lymphoblastic leukemia, acute myeloid leukemia, chronic lymphocytic leukemia, breast cancer, liver cancer, lung cancer, pancreas cancer, B-cell lymphoma, medulloblastoma, and pilocytic astrocytoma). We randomly selected 44 tumors (≈10%) as a test set. Due to the larger cohort, we could decompose the tumors into 15 signatures.

**Fig. 4:**
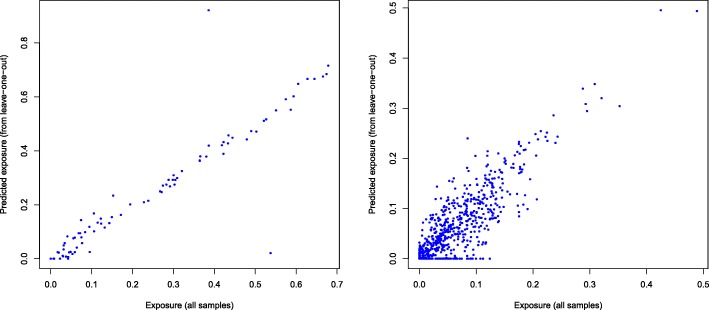
Evaluation of decompTumor2Sig. Comparison of the contributions/weights (“exposures”) predicted for individual tumors (decompTumor2Sig; y-axis) and the collectively computed “true” exposures (pmsignature; x-axis). Left panel: leave-one-out test on 21 breast cancers (*r*=0.923); right panel: test set of 44 out of 435 tumors (*r*=0.807)

Figure 4 compares the weights predicted for individual tumors (decompTumor2Sig) to those computed using the entire set of tumors (pmsignature). Interestingly, in the leave-one-out cross validation using 21 tumors, we obtained two extreme outliers with a highly discordant prediction (see left panel of Fig. 4). We found these to be predictions for a hyper-mutated sample (PD4120a; 33,073 somatic SNVs within genomic regions with a defined transcription strand, as opposed to a median of 2334 for the remaining tumors). Removing this tumor for the leave-one-out test significantly affects the signatures derived from the remaining tumors and hence the contributions/weights predicted for the hyper-mutated tumor.

Both tests demonstrate a good prediction performance, yielding a generally high concordance between predicted and “true” weights. For the leave-one-out test on 21 breast cancers, the median deviation (absolute difference) of predicted and expected weights is 0.018 (mean: 0.0317 including and 0.0197 excluding the outliers); for the 44 out of 435 tumors of different cancer types, the median deviation is 0.0187 (mean: 0.0262, max: 0.155).

**Fig. 5:**
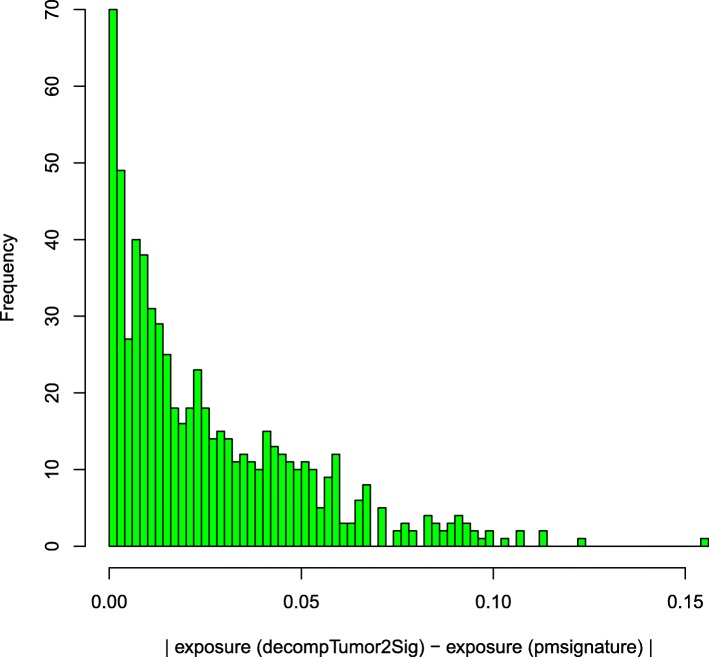
Evaluation of decompTumor2Sig. Absolute differences between the exposures to 15 signatures predicted by decompTumor2Sig for 44 out of 435 tumors of different cancer types, and the respective exposures computed with pmsignature using the full dataset

Figure 5 illustrates that the majority of exposure deviations for the 44 out of 435 tumors of different cancer types tends indeed to be very small. As can be seen, 90% of the deviations are smaller than 0.06, and 99% are smaller than 0.1.

### Simulated genomes

In addition to the evaluation based on experimental data from real tumors, we tested our tool on simulated tumor data using the 15 signatures obtained from the 435 cancers as a starting point for the construction of simulated genomes:

- Each of the 15 signatures was used as a driving signature to generate 1000 simulated genomes: the exposure (or contribution) of the driving signature was set to 80% (=0.8), and the remaining 20% were distributed uniformly among the other signatures.
- Each possible combination of two signatures was used as driving signatures to generate 1000 simulated genomes: the exposures of the driving signatures were set to 50% and 30%, and the remaining 20% were distributed uniformly among the other signatures.
- Each possible combination of three signatures was used as driving signatures to generate 1000 simulated genomes: the exposures of the driving signatures were set to 40%, 25% and 15%, and the remaining 20% were distributed uniformly among the other signatures.
- Each possible combination of four signatures was used as driving signatures to generate 1000 simulated genomes: the exposures of the driving signatures were set to 30%, 20%, 10% and 10%, and the remaining 30% were distributed uniformly among the other signatures.

For each simulated genome, a set of mutations was randomly assigned to the signatures according to the probabilities defined by their simulated contributions. Finally, each simulated genome was decomposed with decompTumor2Sig and the deviation (absolute difference) of the predicted exposures to those set for both the driving signatures and the remaining signatures was verified.

Since for tumors with low mutation burdens the decomposition tends to be more affected by stochastic mutational or systematic sampling noise, we tested different numbers of mutations for the construction of the simulated genomes:

- The median number of mutations in the 21 breast cancer genomes (considering only mutations in genomic regions with a defined transcription strand): 2334 mutations.
- A representative mutation count for the larger set of 435 tumors of various cancer types: 770 mutations, which correspond approximately to the mode, or highest peak, of the log-scaled distribution of the mutation counts (considering only genomic regions with a defined transcription strand) shown Figure S1 in Additional file 1.
- Finally, 200 mutations and 100 mutations, because for low mutation counts noise levels are naturally higher due to the random assignment to mutational signatures.

**Fig. 6:**
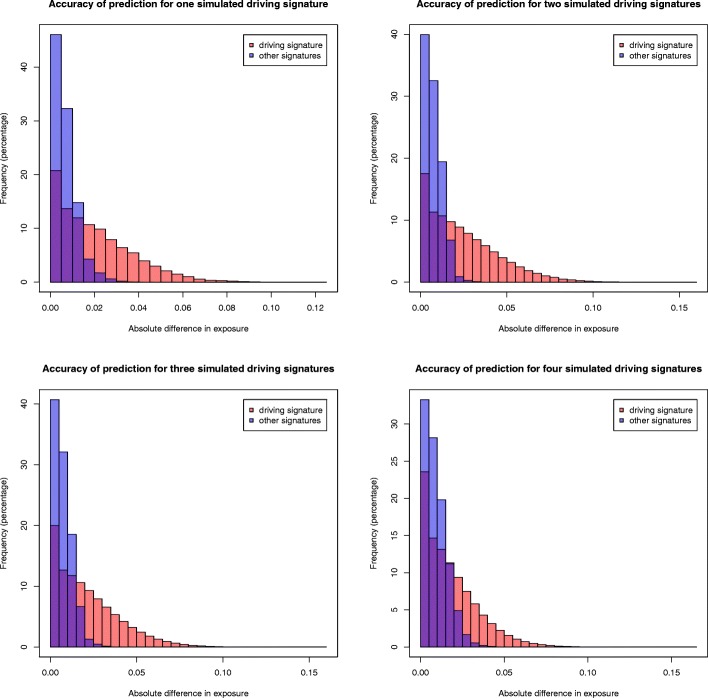
Evaluation based on simulated genomes. Deviations from the expected exposures for simulated genomes with 200 mutations and different numbers of driving signatures: one signature at 80% (upper left), two signatures at 50% and 30% (upper right), three signatures at 40%, 25% and 15% (lower left), four signatures at 30%, 20%, 10% and 10% (lower right)

In all the simulations, most deviations from the expected exposures were negligible, capturing well both the high contributions of the driving signatures and the low contributions of the remaining signatures. We show this exemplarily for 200 mutations in Fig. 6. All other figures can be found in Additional file 1 (Figures S2 to S4). As expected, for higher mutation counts the results are less spurious. For 2334 mutations, for example, most predicted exposures of the driving signatures fall within 2% of the expected value, and next to all exposures of the low contributing signatures within 1% of the expected value. But even for a low mutation count of 200, where both the construction of simulated genomes and their decomposition entail higher noise levels, most of the predictions stay within 5% of the expected value for the high contributing driving signatures and within 2% of the remaining, low contributing signatures (see Fig. 6). The results for 100 mutations are less accurate but still within reasonable bounds.

### Identification of the most relevant signatures

One problem for an accurate decomposition of tumor genomes is that with an increasing number of signatures, the predictions become more spurious. (Likewise, the original construction of the signatures becomes less reliable.) For comparison, we have evaluated the decomposition of 44 out of 435 tumor genomes with 27 instead of 15 signatures, since Shiraishi et al. have proposed this number of signatures in their original paper [3]. (In practice, however, Shiraishi et al. have determined a smaller number of signatures for individual tumor types and then merged the results to obtain a final set of 27 signatures).

The predicted exposures were less accurate with respect to the true exposures than for 15 signatures (see Figure S5 in Additional file 1 and compare to the right panel of Fig. 4).

**Fig. 7:**
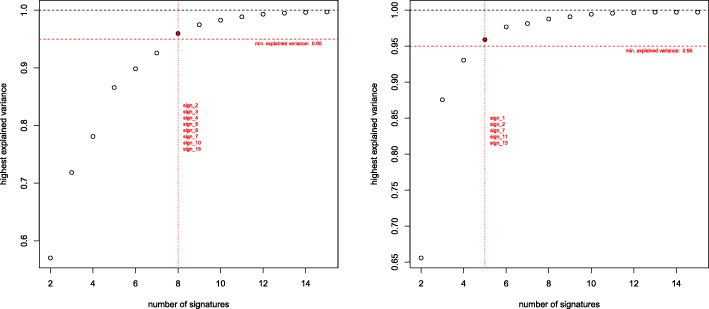
Dependence of explained variance on the number of signatures. For two individual tumors, the fraction of explained variance is determined as a function of the size of the subset of signatures. For each subset size, the set of signatures that achieved the highest fraction of explained variance is plotted. The highlighted signatures are the smallest combinations which can explain ≥95% of the variance

Therefore, we implemented and evaluated a possibility to identify the smallest subset of signatures that can explain a given fraction of the variance of the parameters that describe the mutational patterns of a tumor genome. Figure 7 exemplifies that often already small subsets of signatures can explain the majority of the variance.

**Fig. 8:**
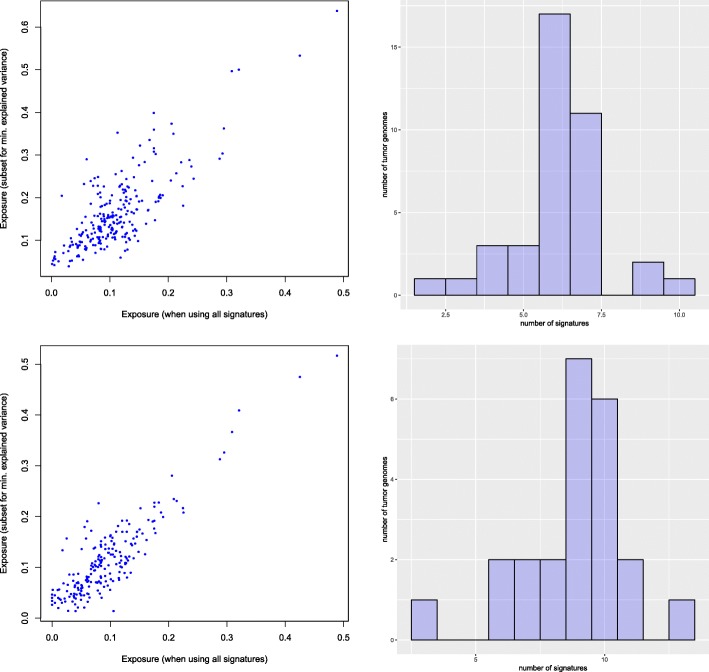
Evaluation of subsets of signatures. Comparison of exposures predicted for subsets of signatures with at least 90% (top) and 97.5% (bottom) explained variance. The scatterplots (left) illustrate the accuracy of the predictions compared to those obtained for all 15 signatures (*r*=0.787 for 90% and *r*=0.884 for 97.5%). The histograms (right) show the distribution of the number of signatures required for these thresholds (*n*=39 for 90% and *n*=23 for 97.5%)

**Fig. 9:**
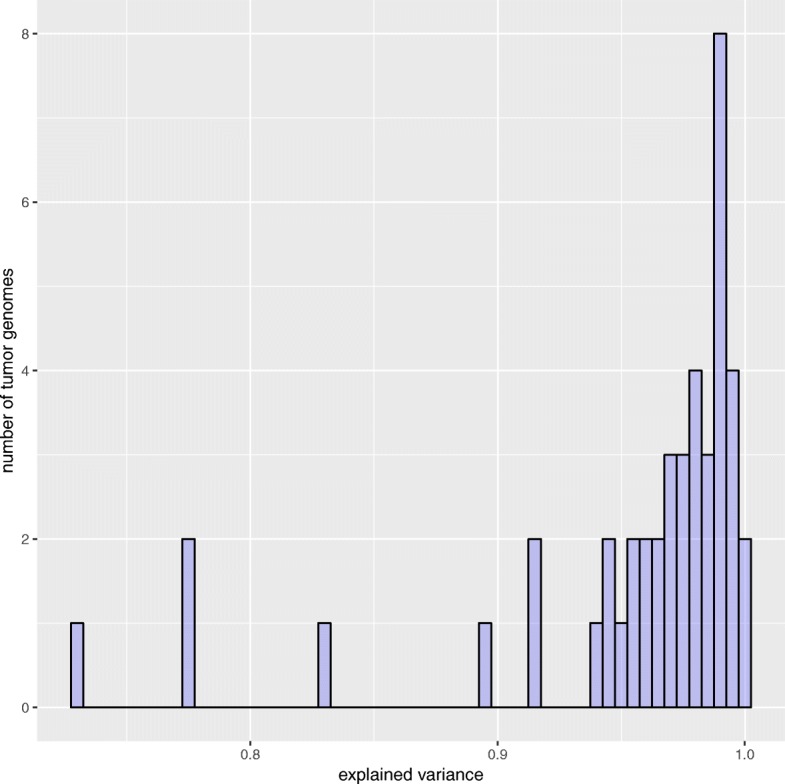
Maximum explained variance. The histogram shows the maximum variance of the mutation patterns of 44 tumor genomes that could be explained when taking all 15 signatures

To evaluate whether the exposures predicted for subsets of signatures can still describe the tumor genomes, we computed the predictions obtained for different thresholds of minimum explained variance. For each of 44 tumor genomes we compared the exposures obtained for the reduced subset of signatures with the exposures which we obtained for the same signatures when using all 15 signatures for tumor decomposition. Figure 8 shows the comparison for two thresholds, 90% and 97.5% of variance explained. For achieving 90% of the explained variance, for example, in most cases seven or less signatures are sufficient. It should, however, be noted that not for all of the 44 tumors such high fractions of explained variance could be reached, but as illustrated in Fig. 9, generally the variance of the mutation patterns of tumor genomes could be explained well.

### Comparison of exposures from refitting and de novo inference

**Fig. 10:**
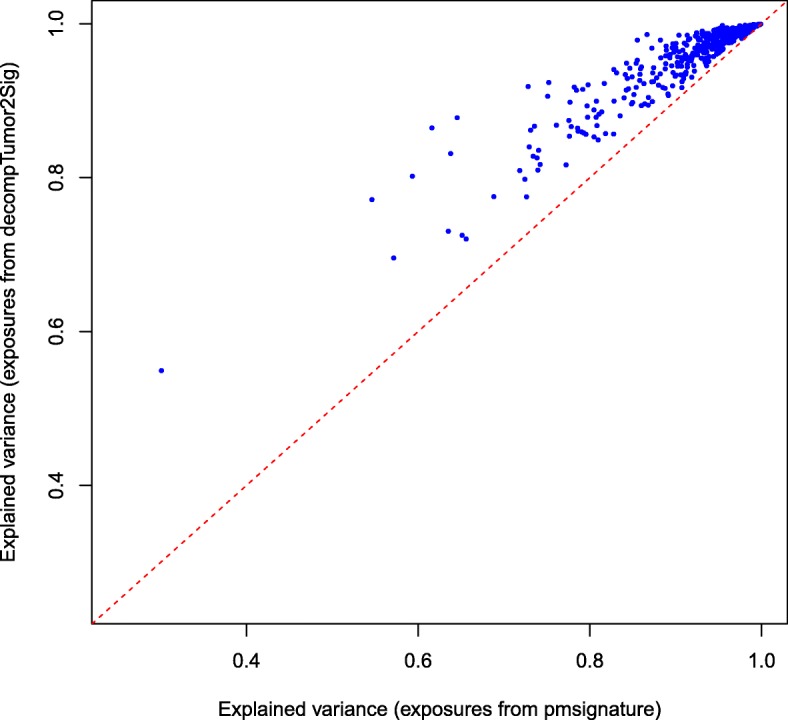
Comparison of signature refitting versus de novo inference. The figure plots for all 435 tumors the explained variance of the exposures as predicted by decompTumor2Sig and originally determined by pmsignature

As already justified above, our evaluation procedure actually underestimates the accuracy of the predicted exposures. This can be shown when analyzing how well the exposures obtained from signature refitting (decompTumor2Sig) explain the variance of the mutation frequencies of all 435 tumors, and directly comparing the variance that can be explained when taking the exposures originally obtained from the de novo signature inference (pmsignature). Figure 10 proves that the exposures obtained from signature refitting are indeed more accurate than those originally obtained from the signature inference with pmsignature.

### decompTumor2Sig for Alexandrov-type signatures

**Fig. 11:**
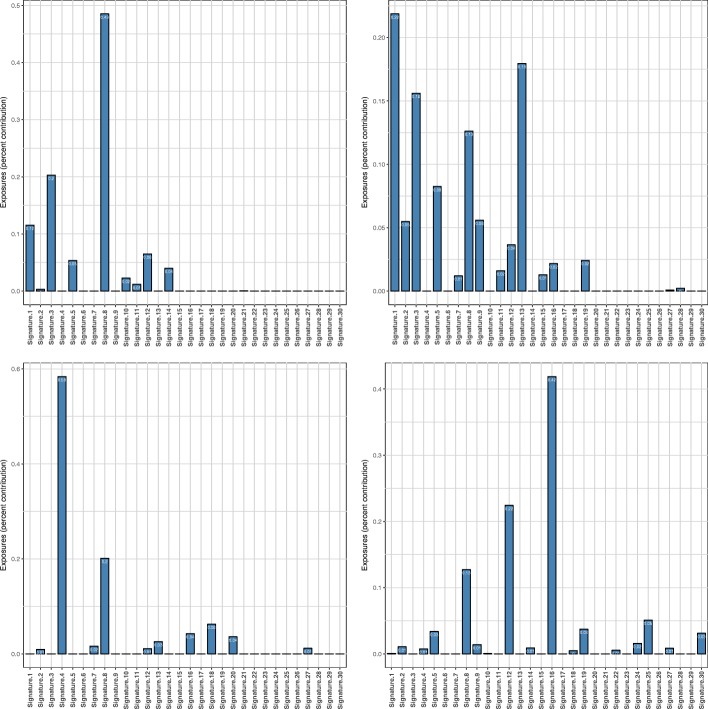
Decomposition into Alexandrov signatures. Contributions of 30 Alexandrov signatures from COSMIC for four tumor genomes: medulloblastoma MB92 (upper left), breast cancer PD4267a (upper right), lung adenocarcinoma LUAD-S01302 (lower left), and liver cancer RK010_C01 (lower right)

Although this article discusses mostly Shiraishi-type signatures, decompTumor2Sig can also be used to quantify the contributions of, or exposures to, Alexandrov-type signatures using the same function calls, as described above. Figure 11 shows examples for four different tumor types. The lung adenocarcinoma, for instance, is strongly characterized by Alexandrov signature 4, which is most likely associated with tobacco smoking [2]. Also the other three examples show significant contributions of signatures that have been found in many tumors of the respective tumor types (see supplementary material to [2]).

### decompTumor2Sig for converted Alexandrov-type signatures

**Fig. 12:**
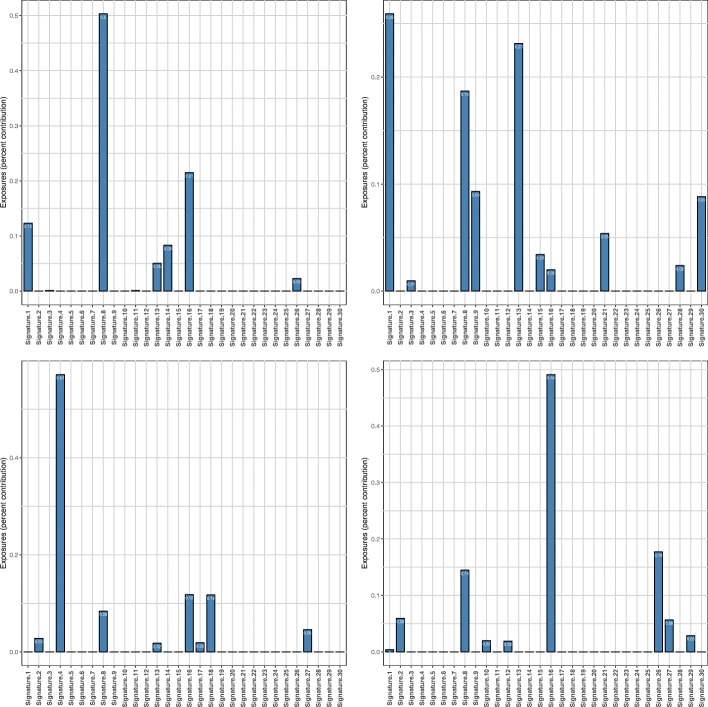
Decomposition into converted Alexandrov signatures. Contributions of Shiraishi signatures—obtained by converting the original 30 Alexandrov signatures from COSMIC—to the four tumor genomes shown in Fig. 11 (same order as there)

**Fig. 13:**
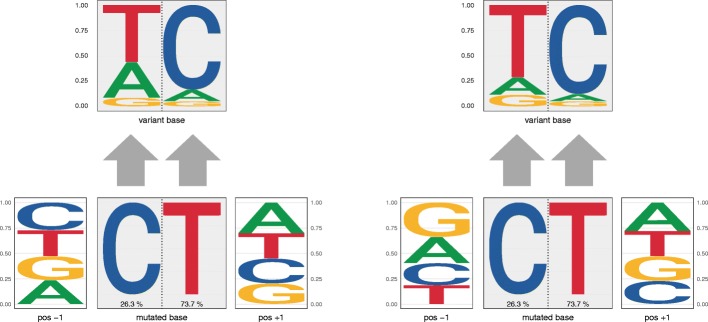
Specificity loss due to conversion of Alexandrov signatures. COSMIC signatures 12 (left) and 26 (right) after conversion from the Alexandrov to the Shiraishi format. While the two signatures are highly similar already in the Alexandrov format (see https://cancer.sanger.ac.uk/cosmic/signatures), after conversion they are nearly indistinguishable

Finally, we illustrate the decomposition of the same four tumors shown in Fig. 11 using not the original Alexandrov-type signatures from COSMIC, but their respective Shiraishi-type signatures obtained with convertAlexandrov2Shiraishi (see Implementation). In three out of four cases, the estimated exposures obtained for the converted signatures (see Fig. 12) have a strikingly high concordance with those presented in Fig. 11. For the lung adenocarcinoma (lower left panel), for example, changes in estimated exposures are observed mostly for marginally contributing signatures. For the medulloblastoma (upper left panel) and the liver cancer (lower right panel), slightly more pronounced changes can be observed, because in both cases the second most contributing signature changes: for the medulloblastoma COSMIC signature 3 is substituted by the converted signature 16, and for the liver cancer COSMIC signature 12 is substituted by converted signature 26. In both cases this can be explained by the fact that after the conversion, these signatures are very similar to each other (Frobenius distance of 0.30 and 0.17, respectively, while the average pairwise Frobenius distance is 0.83 with a standard deviation of 0.29). The conversion of COSMIC signatures 12 and 26, for example, constitutes a particularly evident loss of specificity. As illustrated in Fig. 13, after conversion the two signatures are nearly identical, justifying that one is substituted for the other when decomposing the liver cancer genome. A similar case can be made for COSMIC signatures 3 and 16 and their substitution for the medulloblastoma (not shown). For more complex tumor genomes, as the breast cancer in the upper right panels of Figs. 11 and 12, the exposure prediction after conversion is more divergent. Nevertheless, the exposures of many of the most important signatures are still predicted quite well (namely signatures 1, 8, 9 and 13).

### Mapping between Shiraishi and Alexandrov signatures

When converting the thirty Alexandrov-type signatures from COSMIC to the Shiraishi model, and downgrading our fifteen Shiraishi-type signatures to sequence triplets without transcription-strand information, we can observe, for example, the best match for the signature shown in Fig. 2 to be, as expected, the COSMIC signature for age-related spontaneous deamination of 5-methylcytosine of Fig. 1.

## Conclusions

We have implemented a tool for dissecting mutational catalogs of individual tumor samples in terms of both the simplified mutational signature model proposed by Shiraishi et al. [3] and the full mutational signature model proposed by Alexandrov et al. [1, 2]. The tool is provided as a user-friendly R package, decompTumor2Sig, and shows a good performance as illustrated by the examples we have discussed in this article.

## Additional file

Additional file 1 Supplementary Figure S1: Distribution of the number of mutations in regions with a defined transcription strand of the 435 cancers of various types. Supplementary Figures S2-S4: Evaluation based on simulated genomes. Deviations from the expected exposures for simulated genomes with 100, 770 or 2334 mutations and different numbers of driving signatures. Supplementary figure S5: Comparison of contributions/weights (“exposures”) predicted for individual tumors and collectively computed “true” exposures using 27 signatures instead of 15. Supplementary figure S6: Mutational signature for mutations caused by spontaneous deamination of 5-methylcytosine according to the Shiraishi model and displayed using pmsignature. (PDF 120 kB)

## Abbreviations

A: adenine
C: cytosine
COSMIC: Catalogue Of Somatic Mutations In Cancer [13]
CpG: cytosine-guanine dinucleotide
DNA: deoxyribonucleic acid
G: guanine
MPF: Mutation Position Format
RSS: residual sum of squares
SNV: single nucleotide variant
T: thymine
UV: ultraviolet
VCF: Variant Call Format
