# Supplementary material for: decompTumor2Sig: identification of mutational signatures active in individual tumors
Source: BMC Bioinformatics. 2019 Apr 18;20(Suppl 4):152. doi: 10.1186/s12859-019-2688-6 (PMC6472187; doi:10.1186/s12859-019-2688-6)

# Supplementary material to: decompTumor2Sig: Identification of mutational signatures active in individual tumors

Sandra Krüger, Rosario M. Piro

## Supplementary Figure S1

Distribution of the number of mutations in regions with a defined transcription strand of the 435 cancers of various types.

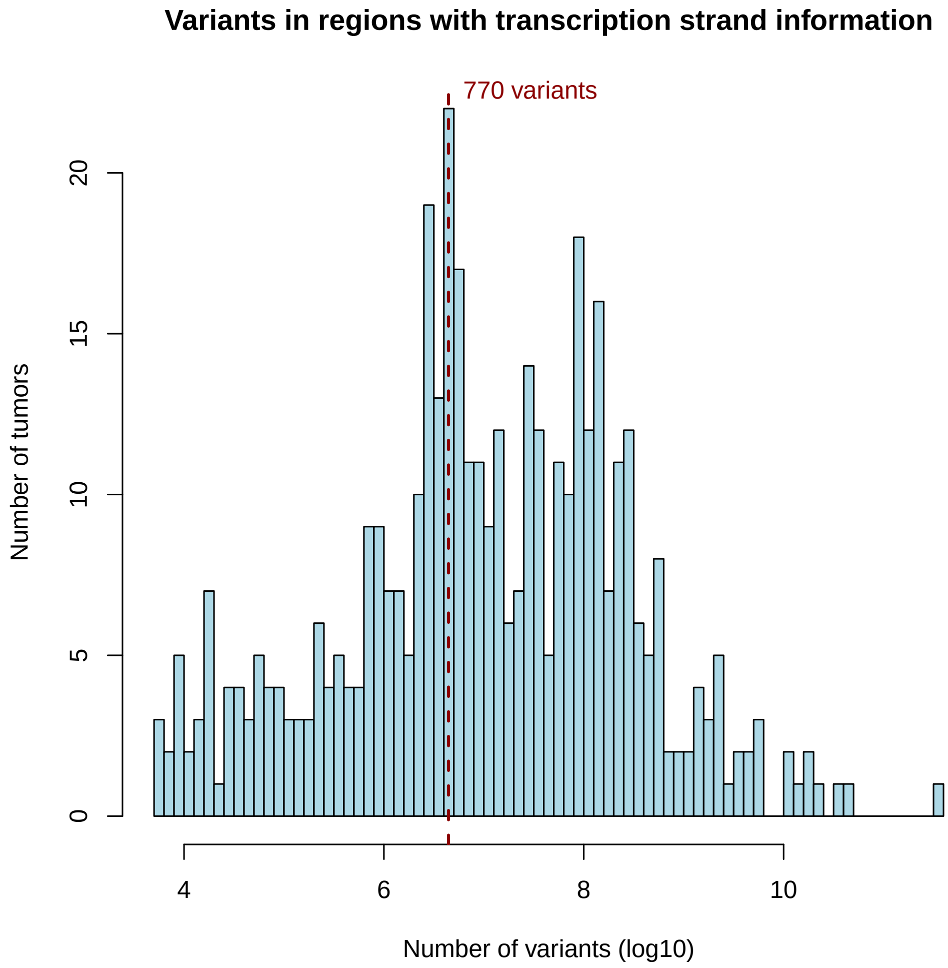

# Supplementary Figure S2

Evaluation based on simulated genomes. Deviations from the expected exposures for simulated genomes with 100 mutations and different numbers of driving signatures: one signature at 80% (upper left), two signatures at 50% and 30% (upper right), three signatures at 40%, 25% and 15% (lower left), four signatures at 30%, 20%, 10% and 10% (lower right).

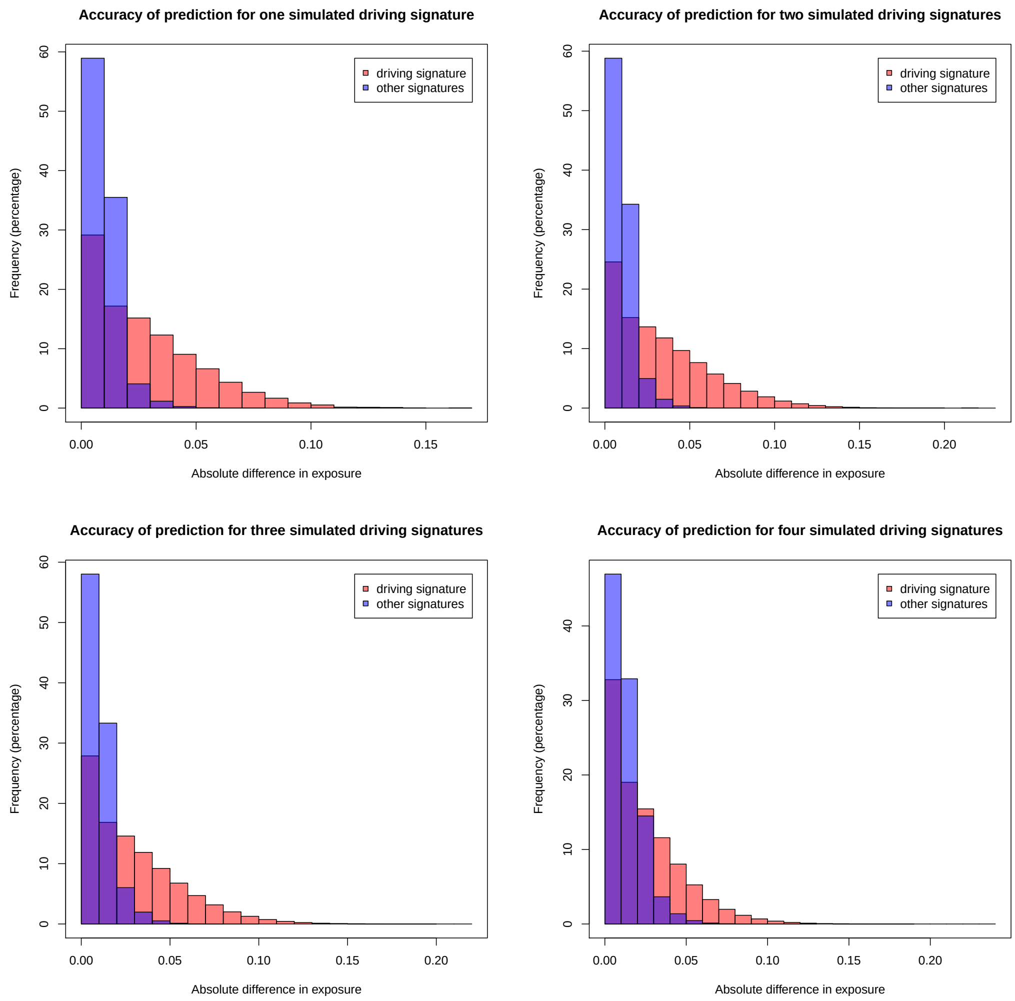

# Supplementary Figure S3

Evaluation based on simulated genomes. Deviations from the expected exposures for simulated genomes with 770 mutations and different numbers of driving signatures: one signature at 80% (upper left), two signatures at 50% and 30% (upper right), three signatures at 40%, 25% and 15% (lower left), four signatures at 30%, 20%, 10% and 10% (lower right).

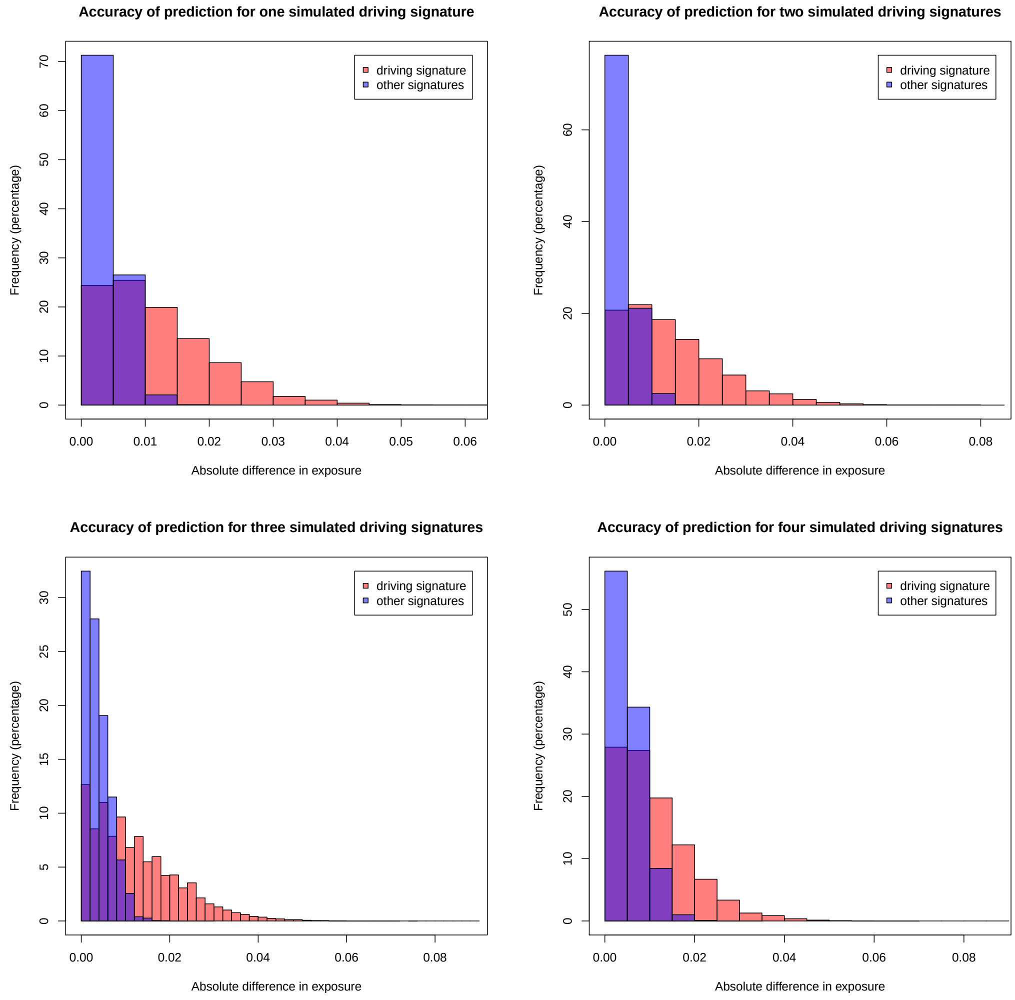

# Supplementary Figure S4

Evaluation based on simulated genomes. Deviations from the expected exposures for simulated genomes with 2334 mutations and different numbers of driving signatures: one signature at 80% (upper left), two signatures at 50% and 30% (upper right), three signatures at 40%, 25% and 15% (lower left), four signatures at 30%, 20%, 10% and 10% (lower right).

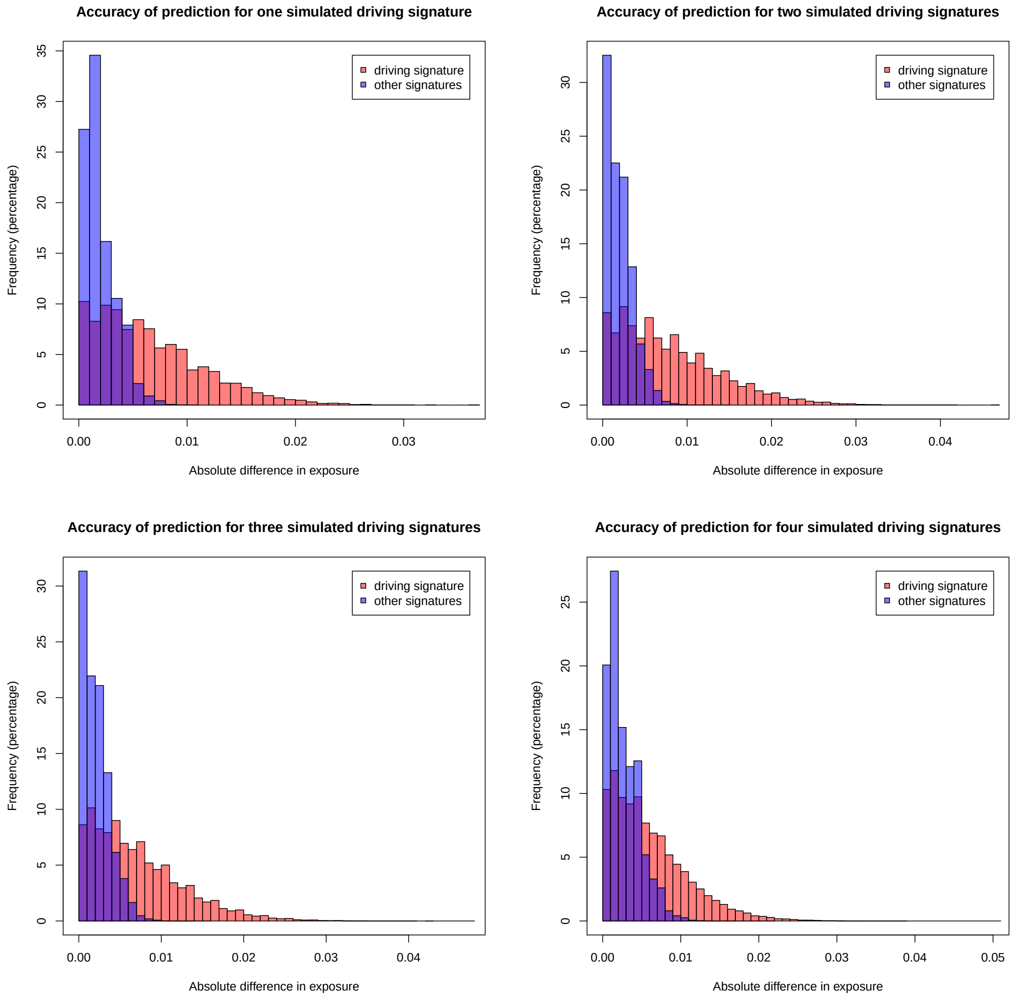

## Supplementary Figure S5

Comparison of contributions/weights ("exposures") predicted for individual tumors (decompTumor2Sig; y-axis) and collectively computed "true" exposures (pmsignature; x-axis). Data: as in the right panel of Fig. 4 of the paper, but using 27 signatures instead of 15.

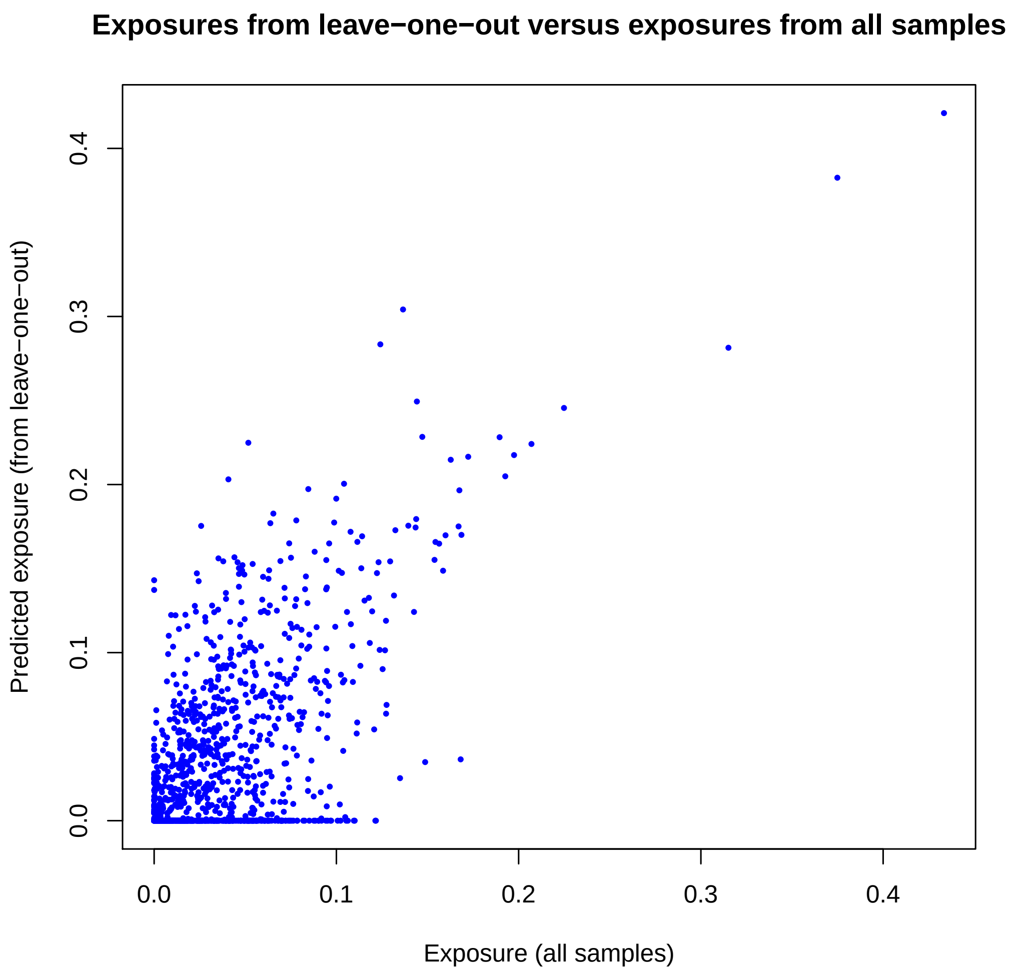

# Supplementary Figure S6

Mutational signature for mutations caused by spontaneous deamination of 5-methylcytosine according to the Shiraishi model and displayed using `pmsignature`. Compare this figure to Fig. 2 in our paper, where the same signature data is plotted using `decompTumor2Sig`.

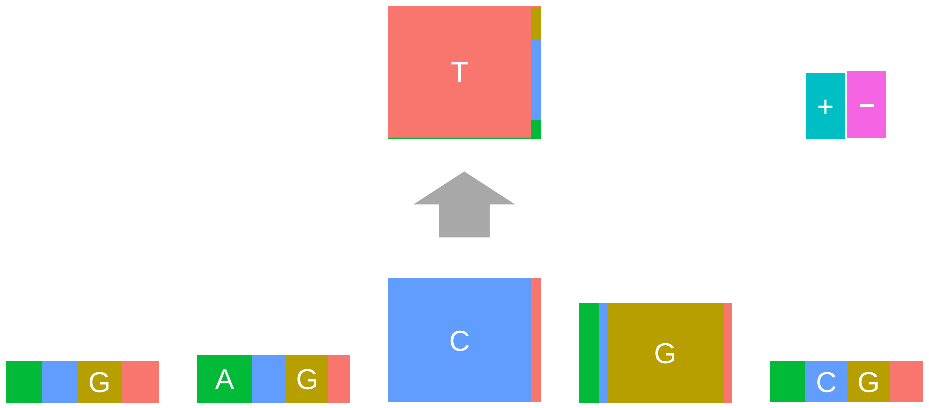

Supplement: Supplementary file 1 — Supplementary Figure S1: Distribution of the number of mutations in regions with a defined transcription strand of the 435 cancers of various types. Supplementary Figures S2-S4: Evaluation based on simulated genomes. Deviations from the expected exposures for simulated genomes with 100, 770 or 2334 mutations and different numbers of driving signatures. Supplementary figure S5: Comparison of contributions/weights (“exposures”) predicted for individual tumors and collectively computed “true” exposures using 27 signatures instead of 15. Supplementary figure S6: Mutational signature for mutations caused by spontaneous deamination of 5-methylcytosine according to the Shiraishi model and displayed using pmsignature. (PDF 120 kB) [file 12859_2019_2688_MOESM1_ESM.pdf]
